# Paroxysmal Atrial Fibrillation as the First Presentation of Coronary Artery Spasm

**DOI:** 10.7759/cureus.71186

**Published:** 2024-10-10

**Authors:** Bahaael Din El Sady, Ahmed M Shaker, Ahmed Abdulsaboor, Assem Ahmed, Abou Bakr M Salama

**Affiliations:** 1 Anesthesiology, Great Western Hospital, NHS Foundation Trust, Swindon, GBR; 2 Physiology, National Research Centre, Cairo, EGY; 3 Clinical Pathology, Faculty of Medicine, Zagazig University, Zagazig, EGY; 4 Emergency, Basingstoke and North Hampshire Hospital, Basingstoke, GBR; 5 Surgery, Baylor College of Medicine, Houston, USA

**Keywords:** calcium channel blockers, cardiology interest, case report, coronary spasm, paroxysmal atrial fibrillation

## Abstract

Atrial fibrillation (AF) is a common arrhythmia with increasing incidence and prevalence, associated with increased morbidity and mortality. The list of predisposing factors is extensive and includes coronary artery disease (CAD) as a significant trigger. Coronary spasm, an uncommon form of CAD, can very rarely present with AF. We report an adult male with no significant risk factors or family history, except for smoking, who presented with recurrent palpitations diagnosed and managed as paroxysmal AF. Within one year, the patient experienced recurrent attacks of acute chest pain associated with paroxysms of AF. Coronary angiography showed normal coronary anatomy, and coronary spasm was confirmed by a provocation test, which was associated with the development of arrhythmia. The calcium channel blocker (CCB) nifedipine and isosorbide mononitrate prevented the recurrence of these attacks. CAD should be managed as part of the holistic management of paroxysmal AF, and coronary artery spasms should be considered and treated with coronary dilators, especially CCBs.

## Introduction

Atrial fibrillation (AF) is one of the most common supra-ventricular arrhythmias with an anticipated increase in the incidence and prevalence rates [1]. It is associated with an increased risk of all-cause mortality [2]. Recurrent paroxysms of symptomatic AF warrant investigation of possible underlying causes including thyroid function abnormalities, electrolyte disturbances, and ischemic heart disease for long-term rhythm reversion [3]. Coronary artery spasm (CAS) is a frequent cause of chest pain. It is sometimes associated with ST elevation or cardiac troponin elevation [4]. Usually, chest pain is the most common presenting symptom for CAS [4]. CAS has been reported during radiofrequency ablation for AF treatment; however, there are no reports of paroxysmal AF as the presenting symptom for CAS [5-7].

## Case presentation

A 32-year-old male presented to the emergency room (ER) with irregular palpitations, primarily triggered by physical and mental stress. The patient had similar attacks previously that had a slower rate and were self-limited. His past medical history is unremarkable with a negative family history. He was a New York Heart Association (NYHA) functional class I and a current smoker.

On physical examination, he was of average build and weight. He was fully conscious with no significant clinical findings but for the irregular rapid pulse (apical heart rate was around 160 beats per minute). Otherwise, the rest of the vital signs were within normal ranges. Echocardiography showed average dimensions of all heart chambers, normal wall thickness, and normal functions. All valves had normal morphology and no significant structural abnormalities.

The patient was medically cardioverted with propafenone according to the hospital protocols. After restoring the sinus rhythm, electrocardiography, and repeated echocardiography were unremarkable and all routine laboratory findings were within accepted range, so the patient was discharged and instructed on the use of the anti-arrhythmic propafenone during the attack.

Over the next few months, the patient was admitted six times for recurrent paroxysms of AF, each triggered and presenting in the same way, requiring medical cardioversion. He was kept on different regimens of amiodarone, sotalol, or propafenone to maintain the sinus rhythm but in vain. The workup for possible triggering factors, including kidney function tests, thyroid function tests, electrolytes panel, pulmonary functions, and sleep apnea investigation, was negative.

One year after the first presentation, the patient complained of typical chest pain, which was precipitated by physical and mental stress and relieved by rest and vasodilators (namely, nitrates). The attack was followed by the development of irregular palpitations. The patient presented to the emergency department (ED), and the paroxysmal AF was electrically cardioverted. The patient presented to our facility over the following three months, and coronary angiography was performed, revealing normal coronary artery anatomy (Videos 1-2).

**Video 1:** Catheter angiography of the right coronary artery showing normal right coronary anatomy.

**Video 2:** Catheter angiography of the left coronary system showing normal anatomy.

During the procedure, we performed a hyperventilation provocation test for coronary spasm, and we documented the right coronary artery spasm as shown by the lack of wash of the contrast until the catheter was disengaged (Video 3).

**Video 3:** Hyperventilation during catheterization resulted in a spasm of the right coronary artery, evidenced by a lack of washout of the angiographic contrast until the catheter was disengaged.

The spasm was associated with chest pain and the development of atrial tachycardia and AF. The patient received intra-coronary nitrates, the spasm was relieved, and AF was restored to sinus rhythm.

In the following months, the patient was maintained on a combination of isosorbide mononitrate 40 mg and nifedipine 30 mg once daily, while also being advised to refrain from smoking. Keeping on the prescribed medications, the patient reported no palpitations or chest pain for one year of follow-up at the outpatient clinic. Holter monitoring was negative for silent AF.

## Discussion

AF is a common atrial tachycardia with a high global burden. It is estimated that 3% of adults over 20 years of age have AF, with a higher prevalence in older populations [1]. Conditions like heart failure, coronary artery disease (CAD), hypertension, structural heart diseases, obesity, obstructive sleep apnea, and chronic kidney disease are associated with a higher risk of AF development [8]. The underlying mechanisms of AF encompass a complex interaction of electrical and structural changes in the atria, often referred to as atrial remodeling. The focal initiation of arrhythmia, particularly from ectopic foci in areas such as the pulmonary veins, triggers the disorganized electrical activity characteristic of AF. This process is further complicated by the development of rotors - spiral wave reentries that propagate chaotic electrical impulses throughout the atria [9]. In addition to these electrical abnormalities, alterations in ion channel function contribute to AF by disrupting normal action potential propagation, particularly through changes in calcium handling and potassium channel activity. This contributes to shortened atrial refractoriness, promoting the persistence of AF [10].

Atrial remodeling involves both electrical and structural changes. Electrical remodeling includes alterations in ion channel expression and function, which facilitate AF maintenance. Structural remodeling involves changes like atrial fibrosis, dilation, and extracellular matrix deposition, creating a substrate that supports the continuation of abnormal electrical activity. Both forms of remodeling are often driven by factors such as inflammation, oxidative stress, and atrial stretch, creating a vicious cycle where AF promotes further atrial remodeling, perpetuating the arrhythmia [11]. Together, these mechanisms create a highly dynamic and multifactorial process that supports the initiation, maintenance, and progression of AF [12].

AF is associated with increased morbidity and mortality, recurrent hospitalizations, and poor quality of life. Reports suggest men with AF have a 1.5-fold increase in all-cause mortality, while the risk increases twofold in females [1]. The most common preventable complication of AF is stroke [8]. Symptoms vary widely with some patients being completely asymptomatic to severe disabling symptoms. Prompt diagnosis and evidence-based management reduce AF-associated morbidities and mortality. AF treatment focuses on rate control, rhythm control, and stroke prevention. Rate control is achieved through medications like beta-blockers, calcium channel blockers (CCBs), or digoxin to manage heart rate without restoring normal rhythm. For rhythm control, antiarrhythmic drugs such as amiodarone or flecainide, electrical cardioversion, or catheter ablation, especially pulmonary vein isolation, are employed to restore and maintain sinus rhythm. Stroke prevention is critical, and anticoagulation therapy using warfarin or direct oral anticoagulants (DOACs) like apixaban or rivaroxaban is recommended to reduce the risk of thromboembolism. Additionally, lifestyle modifications, including weight management and treating underlying conditions such as hypertension or sleep apnea, are important in optimizing AF treatment outcomes. Treatment strategies are tailored based on individual patient characteristics and risk factors. Rhythm reversion is recommended for symptom relief. It is advised also to investigate and control predisposing conditions [1,2,13].

CAD is a major risk factor for the development of AF, with a hazard ratio being 1.46 in the case of myocardial infarction [14]. It is advised to control the underlying CAD either medically or interventionally for better AF control. Most studies that address the relationship between CAD and AF development focus on the major causes of myocardial ischemia including obstructive atherosclerotic coronary disease, while less light is being spotted on coronary artery spasms as a cause of paroxysmal AF. Sueda et al. showed that 16.2% of patients who underwent spasm provocation tests developed paroxysmal AF, with the right coronary spasm being more implicated. They suggested autonomic dysregulation plays a role in development of the paroxysmal AF [15].

Provocation tests for coronary spasms include the hyperventilation test, which is the least sensitive but most specific (65% sensitivity and 100% specificity), as well as acetylcholine provocation tests (85% sensitivity and 80% specificity) [16,17]. Medical treatment is controversial to a great extent with coronary dilators, especially CCBs being the cornerstone of treatment. Although nitrates have an immediate vasodilator effect, CCBs have long-acting inhibition of Ca^2+^ inflow into coronary smooth muscles with dramatic efficacy against coronary spasms [18]. Hung et al. reported a similar case of AF paroxysms due to coronary spasms, which responded to nifedipine treatment [10].

Coronary spasm leads to sinoatrial (SA) node ischemia, hindering its maestro role in rhythm control. Ischemia also increases the automaticity of the ectopic foci that will have the upper hand over the SA node, initiating atrial tachycardia that degenerates to AF. Nifedipine regulates the Ca^2+^ currents in the coronary smooth muscles, preventing coronary spasms and minimizing the chances of AF development. A key lesson from this case is that the delayed consideration of CAS as a trigger of paroxysmal AF hindered the adequate management of the case for 12 months. Accordingly, we suggest that CAS should be considered as a part of the holistic work for paroxysmal AF management.

In summary, we report a case of recurrent paroxysmal AF due to coronary spasm, which was provoked in the Cath lab by a hyperventilation test. The rhythm was reverted after intracoronary injection of nitrates. Long-term nifedipine and nitrates treatment eliminated the paroxysms over one year of follow-up. Regular follow-up is planned to confirm the long-term control of the AF paroxysms in this patient. As a report for a single case, we believe more studies are required to consolidate our knowledge about the best management strategies for coronary artery spasm-induced AF.

## Conclusions

Paroxysmal AF is a common presentation in the ER. It may develop secondary to coronary artery spasms, especially spasms in the right coronary artery. Coronary spasms should be considered for management as part of the overall management of AF. Coronary spasms should be suspected in middle-aged patients with no structural heart disease. CCBs together with nitrates provide good control for paroxysmal atrial AF induced by coronary spasm. However, randomized controlled trials to consolidate the management protocols are required.
